# From enhanced collaborations to space advancements: technologies to bring libraries (and librarians) full circle and into the future

**DOI:** 10.5195/jmla.2019.788

**Published:** 2019-10-01

**Authors:** Patricia F. Anderson, Emily J. Hurst

**Affiliations:** Virtual Projects Advisory Committee, and Emerging Technologies Informationist, Taubman Health Sciences Library, University of Michigan, 1135 East Catherine Street, Ann Arbor, MI, 48109, pfa@umich.edu; Virtual Projects Advisory Committee, and Deputy Director/Head, Research and Education, Tompkins-McCaw Library for the Health Sciences, Virginia Commonwealth University Libraries, 509 North 12th Street, Box 980582 Richmond, VA 23298-0582, ejhurst@vcu.edu

## Abstract

Since the *Journal of the Medical Library Association (JMLA)* Virtual Projects section was first announced in 2012, the virtual projects featured in the *JMLA* have expanded or improved library spaces, services, collaborations, connections, and future directions. Virtual projects selected by the *JMLA* Virtual Projects Section Advisory Committee have been both practical and responsive to library and patron needs and illustrate ways that librarians are leading their communities and services in new directions. Virtual projects highlighted in this year’s section demonstrate innovative adaptations of technology into the modern medical library that strengthen collaborative commitments and clinical and research partnerships. They also illustrate how technologies support the idea of “library as place” by providing spaces for users to explore new technologies, as well as tools for space and service planning. This year’s virtual projects fully embrace changes in learning, research patterns, technologies, and the role of the health sciences librarian and the library.

The Virtual Projects column in the *Journal of the Medical Library Association (JMLA)* was first announced in 2012, with the original vision offering a parallel to the building projects column, “since so much construction now involves websites rather than buildings” [1]. In the past seven years, the column has presented many new technologies that have been adopted by librarians, including projects incorporating new technologies for expanding or improving library spaces, services, collaborations, connections, and future directions. In 2018, the Virtual Projects Section evolved from a column to a special section, providing more opportunities for libraries to share experiences for incorporating technology. The *JMLA* Virtual Projects Section Advisory Committee encourages submissions that highlight innovative technology solutions in health sciences libraries, selecting both those that are practical and responsive to library and patron needs and others that illustrate ways in which librarians are leading their communities and services in new directions [2].

Technology trends continue to influence health sciences libraries in various ways. As librarians create innovative services and programs, they embrace the potential of new technologies to solve old problems and create new services. This year’s virtual project submissions related to emerging and established technologies, from artificial intelligence to virtual reference, including text mining, research visualizations, open educational resources, and many other projects from both small and large academic and hospital libraries. The highlighted projects in this issue of the *JMLA* demonstrate innovative adaptations of technology into the modern medical library.

In recent years, academic and health librarians have reinvented themselves and have created opportunities as partners and collaborators in spaces that are historically new to the profession. Among these, librarians are increasingly recognized as co-occupants of research spaces, alongside faculty, clinicians, and full-time researchers. Today, libraries regularly hire for positions with titles like research impact librarian, data visualization specialist, data management librarian, and clinical integration specialist, to name just a few.

As the functionality of roles for librarians evolve, the integration of new technologies and services is common. Adopting technologies that strengthen collaborative commitments and clinical and research partnerships is demonstrated by four projects highlighted in this special section:

- “Bibliometric Mapping for Current and Potential Collaboration Detection”
- “Multisite Collaboration Using REDCap to Capture Library Data”
- “Text Mining for Clinical Support”
- “What Can We Do about Dr. Google? Utilizing the Electronic Medical Record (EMR) to Prescribe Reliable Online Patient Education”

What “place” means in the context of modern medical libraries continues to evolve, and technologies enhance the idea of “library as place.” Many physical libraries now provide spaces specifically for making, collaboration, digital access, digital literacy, and education, to name only a few uses. Library spaces continue to evolve, and many of these spaces are enhanced by or with the adoption of technology. Technologies can help with space tracking and planning, providing libraries with data to make more informed decisions related to space and service planning. Libraries also provide havens for medical professionals who are interested in the applied use of virtual reality. Creating spaces for users to explore virtual reality and experiment with this technology is becoming more broadly adopted in libraries. Two projects in this section underscore the importance of technology in enhancing understanding of library space.

- “Creating a New ‘Reality’ for Medical Education: The Nexus Reality Lab for Virtual Reality”
- “Data-Driven Space Planning: Using Suma to Collect Data”

Each year, the technologies highlighted in the *JMLA* Virtual Project section expand the vision of a virtual project. There is a profound sense of growth and future possibilities in witnessing how health sciences librarianship adopts different technologies as the profession evolves. This year’s virtual projects fully embrace changes in learning, research patterns, technologies, and the role of the health sciences librarian and the library.

Material for this year’s column was selected with the assistance of the *JMLA* Virtual Projects Advisory Committee: Patricia F. Anderson; Emily J. Hurst, AHIP; Michelle Kraft, AHIP, section coeditor; Susan Lessick, AHIP, FMLA, section editor; J. Dale Prince, AHIP; and Elizabeth C. Whipple, AHIP. Selected projects were edited by Susan Lessick, AHIP, FMLA and coedited by Michelle Kraft, AHIP.

**Patricia F. Anderson, MILS**, pfa@umich.edu, https://orcid.org/0000-0001-6348-2324, Virtual Projects Advisory Committee, and Emerging Technologies Informationist, Taubman Health Sciences Library, University of Michigan, 1135 East Catherine Street, Ann Arbor, MI, 48109

**Emily J. Hurst, MSLS, AHIP**, ejhurst@vcu.edu, https://orcid.org/0000-0003-4191-1938, Virtual Projects Advisory Committee, and Deputy Director/Head, Research and Education, Tompkins-McCaw Library for the Health Sciences, Virginia Commonwealth University Libraries, 509 North 12th Street, Box 980582 Richmond, VA 23298-0582
